# Reinforcement learning of self-regulated β-oscillations for motor restoration in chronic stroke

**DOI:** 10.3389/fnhum.2015.00391

**Published:** 2015-07-03

**Authors:** Georgios Naros, Alireza Gharabaghi

**Affiliations:** ^1^Division of Functional and Restorative Neurosurgery and Division of Translational Neurosurgery, Department of Neurosurgery, Eberhard Karls University TuebingenTuebingen, Germany; ^2^Neuroprosthetics Research Group, Werner Reichardt Centre for Integrative Neuroscience, Eberhard Karls University TuebingenTuebingen, Germany

**Keywords:** reinforcement learning, beta oscillations, brain-robot interface, brain-machine interface, brain-computer interface, hand function, functional restoration, stroke

## Abstract

Neurofeedback training of Motor imagery (MI)-related brain-states with brain-computer/brain-machine interfaces (BCI/BMI) is currently being explored as an experimental intervention prior to standard physiotherapy to improve the motor outcome of stroke rehabilitation. The use of BCI/BMI technology increases the adherence to MI training more efficiently than interventions with sham or no feedback. Moreover, pilot studies suggest that such a *priming* intervention before physiotherapy might—like some brain stimulation techniques—increase the responsiveness of the brain to the subsequent physiotherapy, thereby improving the general clinical outcome. However, there is little evidence up to now that these BCI/BMI-based interventions have achieved *operate conditioning* of specific brain states that facilitate task-specific functional gains beyond the practice of *primed* physiotherapy. In this context, we argue that BCI/BMI technology provides a valuable neurofeedback tool for rehabilitation but needs to aim at physiological features relevant for the targeted behavioral gain. Moreover, this therapeutic intervention has to be informed by concepts of reinforcement learning to develop its full potential. Such a refined neurofeedback approach would need to address the following issues: (1) Defining a physiological feedback target specific to the intended behavioral gain, e.g., β-band oscillations for cortico-muscular communication. This targeted brain state could well be different from the brain state optimal for the neurofeedback task, e.g., α-band oscillations for differentiating MI from rest; (2) Selecting a BCI/BMI classification and thresholding approach on the basis of learning principles, i.e., balancing challenge and reward of the neurofeedback task instead of maximizing the classification accuracy of the difficulty level device; and (3) Adjusting the difficulty level in the course of the training period to account for the cognitive load and the learning experience of the participant. Here, we propose a comprehensive neurofeedback strategy for motor restoration after stroke that addresses these aspects, and provide evidence for the feasibility of the suggested approach by demonstrating that dynamic threshold adaptation based on reinforcement learning may lead to frequency-specific operant conditioning of β-band oscillations paralleled by task-specific motor improvement; a proposal that requires investigation in a larger cohort of stroke patients.

## Introduction

Neurofeedback training of Motor imagery (MI)-related brain-states with brain-computer/brain-machine interfaces (BCI/BMI) is currently being explored as an experimental intervention alternative to or prior to standard physiotherapy to improve the motor outcome of stroke rehabilitation. Using BCI/BMI technology increases the adherence to mental training more efficiently than the same interventions with sham or no feedback. Moreover, first results suggest that such a *priming* intervention before physiotherapy might—like some brain stimulation techniques—increase the responsiveness of the brain for the subsequent physiotherapy, thereby improving the general clinical outcome, i.e., independent of the specific BCI/BMI task (Ramos-Murguialday et al., 2013; Pichiorri et al., 2015). While such a result would be valuable in itself, the physiological foundation of BCI/BMI technology raises hopes that functional gains not yet achieved with enhanced physiotherapy might soon be possible. However, when compared to dose-matched robot-assisted therapy, BCI/BMI interventions achieve, at best, only similar clinical benefits (Ang et al., 2010, 2014).

Interestingly enough, those studies that used BCI/BMI neurofeedback prior to physiotherapy, thereby improving the general motor outcome in comparison to the respective control groups, could not demonstrate a significant improvement in controlling the feedback devices, i.e., lacking a learning progress in the neurofeedback task. More specifically, subacute stroke patients, who participated in twelve BCI sessions, showed no significant changes in their neurofeedback performance (Pichiorri et al., 2015). Similarly, chronic stroke patients who performed a mean of seven and eleven sessions with an arm and hand BMI, respectively, could not increase the brain-controlled movement time of these devices during the respective training periods (Ramos-Murguialday et al., 2013). Only those patients who trained with the BMI devices for longer periods could achieve control rates higher than the starting condition—albeit without presenting a performance curve to be expected for a continuous learning experience (Ramos-Murguialday et al., 2013).

Thus, there is little evidence up to now that in stroke patients BCI/BMI-based interventions have achieved *operate conditioning* of specific brain states that facilitate task-specific functional gains beyond the practice of *primed* physiotherapy or *intensive* robot-assisted rehabilitation.

In this context, we argue that while BCI/BMI technology provides a valuable neurofeedback tool for rehabilitation, it needs to target physiologically relevant features and to be informed by concepts of reinforcement learning and cognitive load theory to develop its full potential (Bauer and Gharabaghi, 2015a,b).

Such a refined neurofeedback approach would need to address the following issues: (1) Defining a physiological feedback target that is specific to the intended behavioral gain, e.g., β-band oscillations for cortico-muscular communication. This targeted brain state might be different from the brain state optimal for the neurofeedback task, e.g., α-band oscillations for differentiating MI from rest; (2) Selecting a BCI/BMI classification and thresholding approach on the basis of learning principles, i.e., balancing challenge and reward of the neurofeedback task instead of maximizing the classification accuracy (CA) of the feedback device; and (3) Adjusting the difficulty level in the course of the training period to account for the learning experience and the cognitive load of the participant.

### Physiological Feedback Target

Brain oscillations in α- (8–12 Hz) and β-frequency (15–35 Hz) bands are modulated during actual and imagined movements. Although showing a highly correlated pattern, they nonetheless serve distinct functional mechanisms (van Wijk et al., 2012; Kilavik et al., 2013; Brinkman et al., 2014). While α-activity gates information by inhibiting task-irrelevant regions (Mazaheri and Jensen, 2010), β-activity mediates the disinhibition of the sensorimotor cortex and the coherent interaction with the muscles (Mima et al., 2000; Kristeva et al., 2007; van Wijk et al., 2012; Kilavik et al., 2013; Aumann and Prut, 2014).

In stroke patients, movement-related β-desynchronization (event-related desynchronization, ERD) in the contralateral primary cortex is compromised in comparison to healthy controls, i.e., the more severe the patient’s motor impairment, the less β-ERD (Rossiter et al., 2014). Although, β-ERD has been shown to be feasible as a control signal in principle (Bai et al., 2008), it remains—particularly in the case of severely affected stroke patients—inferior to α-ERD for classification purposes, e.g., for differentiating movement-related brain states for the control of external devices such as BMI (Gomez-Rodriguez et al., 2011). Recent BMI approaches for stroke rehabilitation therefore used MI-related α-ERD to control orthotic training devices (Buch et al., 2008, 2012; Ang et al., 2010, 2014; Shindo et al., 2011; Ramos-Murguialday et al., 2013). Despite generally promising results, clinical improvements for the severely affected and chronic patient group are still missing (Buch et al., 2012) or are limited with regard to the restoration of relevant hand and finger function (Ramos-Murguialday et al., 2013).

In this context, we argue that the fact that β-oscillations might be less optimal for classification purposes, e.g., for differentiating movement-related brain states in many stroke patients, does not compromise but rather qualifies this physiological marker as a therapeutic target. Here, we see an analogy to the concept of constraint-induced movement therapy in stroke patients, where the affected rather than the healthy body side is trained to facilitate restoration instead of compensation of motor function. We therefore propose that restorative BCI/BMI should follow the therapeutic goal of restoring the sensorimotor loop via improved β-band modulation rather than aiming to train the brain state that enables the patient to control the exercising device best. The latter is a strategy that is implicitly followed when selecting individual frequency bands with best classification properties, i.e., that best separate the rest and the task condition (Pichiorri et al., 2015).

A similar rationale may be applied when considering which brain hemisphere should be trained with neurofeedback. Severely impaired patients are often characterized by movement-related activity shifts to the contralesional hemisphere, particularly in the acute and subacute phase after stroke. However, the physiological role of this shift, i.e., either fascilatory or inhibitory for functional restoration, is still not completely clear (Di Pino et al., 2014). As in the majority of BCI/BMI rehabilitation studies in stroke, we therefore propose the strategy of targeting the ipsilesional hemisphere with the therapeutic intervention as long as sufficient corticospinal connectivity, e.g., probed by motor-evoked potentials following transcranial magnetic stimulation, is still present (Stinear et al., 2007, 2012). Neurofeedback of the ipsilesional hemisphere might result in lower BCI/BMI CA than training the healthy hemisphere. However, on the basis of the theoretical framework explained above, the decreased oscillatory range in the ipsilesional hemisphere should not be considered as a limitation but rather as the actual target of reinforcement learning in this therapeutic setting.

### Cognitive Load of BCI/BMI Neurofeedback

A recent analytic approach has provided a theoretical foundation for estimating the participant’s cognitive resources and the instructional efficacy of neurofeedback on the basis of BCI/BMI performance measures (Bauer and Gharabaghi, 2015a).

By integrating classification theory (Theodoridis and Koutroumbas, 2009) with item response theory (Safrit et al., 1989), the relationship between the classification algorithm of a BCI/BMI and the ability for self-regulation has been described (Bauer and Gharabaghi, 2015a). Moreover, on the basis of the cognitive load theory for instructional design (Sweller, 1994; Schnotz and Kürschner, 2007), the BCI/BMI CA may be interpreted within the framework of neurofeedback training by off-line calculation of the positive rates for different classifiers and thresholds (Bauer and Gharabaghi, 2015a). In this context, different performance measures provide information about the subject’s ability and his/her performance when support is provided as well as an indirect measure of the subject’s cognitive resources for coping with the mental load that occurs during a misalignment between ability and difficulty (Allal and Pelgrims Ducrey, 2000; Schnotz and Kürschner, 2007; Bauer and Gharabaghi, 2015a).

Mathematical models and evidence from empirical data suggest that participants with low abilities for brain self-regulation in particular, e.g., stroke patients with a reduced β-band modulation range, might benefit from neurofeedback strategies that take cognitive resources into account and align the ability and task difficulty (Bauer and Gharabaghi, 2015a).

### Reinforcement Learning with BCI/BMI Technology

Due to the treatment rationale of modulating specific brain features, the classifier of restorative BCI/BMI technology is usually constrained, thus posing a particular challenge for the optimization of neurofeedback to the participant, who should be rewarded for achieving this goal (Bauer and Gharabaghi, 2015b). In this context, in which classifiers are often based on linear discriminant analysis, threshold adaptation might be a viable approach to affect reinforcement learning (Theodoridis and Koutroumbas, 2009). Linear methods are characterized by threshold selection, i.e., the definition of a specific value on an one-dimensional continuum spanned between the two states that are to be differentiated (Bauer and Gharabaghi, 2015b). By altering this threshold, the sensitivity and the specificity of the classifier will be modified (Thompson et al., 2013; Bauer and Gharabaghi, 2015b). The selection of this threshold is currently determined by the intent to maximize the CA (Thomas et al., 2013; Thompson et al., 2013). However, mathematical modeling of restorative neurofeedback on the basis of Bayesian simulations indicates that operant conditioning can be optimized when an adaptation strategy for threshold selection is applied in the course of the training (Bauer and Gharabaghi, 2015b). Such an adaptation strategy would need to change the classifier threshold, i.e., difficulty level, of the feedback device to challenge the participant in the course of the training while preserving his/her motivation. Moreover, the provided feedback should retain its specificity and reward trained actions rather than punish false ones (Bauer and Gharabaghi, 2015b).

Here, we propose a comprehensive neurofeedback approach for motor restoration after stroke that addresses these aspects, and provide evidence for the feasibility of the suggested approach by demonstrating that dynamic threshold adaptation based on reinforcement learning may lead to frequency-specific operant conditioning of β-band oscillations paralleled by task-specific motor improvements.

### Adaptation Strategy

The sensitivity and specificity of the classifier of a linear discriminant analysis are indicated by the true-positive rate (TPR) and the true-negative rate (TNR), respectively; the false-positive rate (FPR) equals 1-TNR. TPR and TNR are calculated by

(1) $$TPR\;=\frac{{\text{pN}}_{\text{move}}}{{\text{N}}_{\text{move}}}$$

(2) $$TNR=\frac{{\text{nN}}_{\text{rest}}}{{\text{N}}_{\text{rest}}},$$

with N as the total number of sample blocks in either the rest or move period, and pN and nN as the positively and negatively classified sample blocks, respectively.

The CA of a BMI system is defined by

(3) $$CA=\frac{\text{TPR}+\text{TNR}}{2}.$$

The correct response rate (CRR) is defined as the ratio between the actual occurrence of the anticipated action (e.g., robotic orthosis movement) and the number of trials.

To optimize operant conditioning, we propose a threshold adaptation strategy informed by a Bayesian reinforcement learning model for restorative neurofeedback training (Bauer and Gharabaghi, 2015b). The threshold adaptation strategy is based on the following principles:

1. Facilitating fast and efficient learning by challenging the participant with thresholds of increasing difficulty, i.e., low TPR.
2. Rewarding trained actions rather than punishing false ones by thresholds beyond the maximum CA.
3. Preserving the specificity of the feedback by minimizing the FPR, i.e., maximizing the TNR.
4. Improving the motivation of the participant and the adherence to the training by maximizing the CRR.

The threshold of the linear classifier is adjusted before each neurofeedback session and then kept fixed throughout this session (i.e., training day). This adjustment is conducted on the basis of an offline calculation of the neurofeedback parameters CRR, TPR and TNR/FPR to maximize the following function, which we refer to as the zone of adjusted threshold (ZAT):

(4) ZAT = CRR − (TPR + FPR)

(5) ZAT = CRR − (TPR +(1−TNR)).

## Experimental Setup and Methods

**Figure 1:**
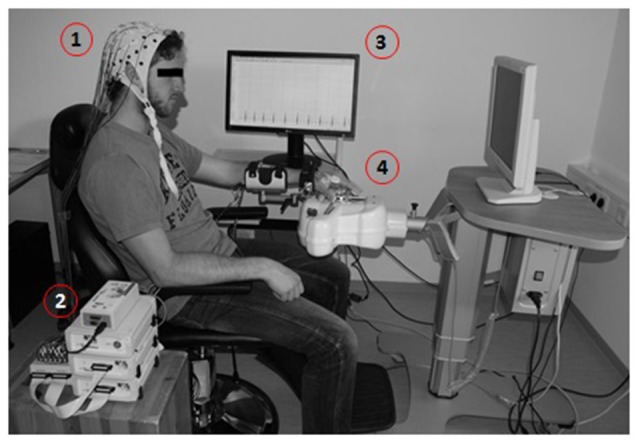
**Brain-machine interface (BMI) training environment presented with a healthy subject.** Motor imagery (MI)-related modulation of oscillatory activity is detected by electroencephalogram, EEG (1), amplified (2) and processed in a BCI2000-based control system (3) to operate a commercially available electromechanical hand orthosis (4).

The BMI-based neurofeedback environment (Figure 1) included a commercially available electromechanical hand orthosis (Amadeo, Tyromotion GmbH, Graz, Austria) which enables mass finger extension and flexion while the wrist remains fixed without any movement. This robotic orthosis is used regularly for standard rehabilitation exercises independent of brain-interfacing. When used in conjunction with BCI/BMI technology, it is also referred to as a brain-robot interface (BRI; Bauer et al., 2015; Vukelić and Gharabaghi, 2015). This BMI/BRI opens and closes the paralyzed hand when triggered by ipsilesional oscillatory brain activity during cued kinesthetic MI which is classified with a linear classifier (Walter et al., 2012; Gharabaghi et al., 2014a). Following each BMI training session, approximately 1 h of goal-oriented physiotherapy was applied by practicing hand opening, finger extension and grasping movements during activities of daily living to enhance the consolidation of the movements trained with BMI feedback.

**Figure 2:**
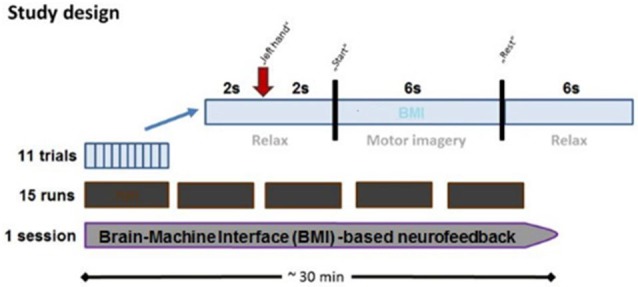
**Time course of BMI session.** Each BMI session lasted approximately 30 min and consisted of fifteen runs separated by short breaks. Every run consisted of 11 trials, each of which lasted 16 s. Each trial started with a rest period (2 s) while subjects were instructed to prepare for MI following an auditory cue (2 s preparation phase), and to imagine the respective reaching movement following a “start” cue (6 s MI phase), which was followed by a “rest” cue (6 s rest phase).

Every session contained 15 runs, each lasting 2–3 min. Every run consisted of 11 trials which began with a 2-s rest period and a preparation period of 2 s, followed by a 6 s movement imagination period and a 6-s rest period (Figure 2). This fixed timing of each trial may lead to anticipation effects, but was intentionally chosen to be comparable with previous BMI studies in healthy subjects and stroke patients.

The onset of the preparation, imagination and rest periods were indicated by the commands “left hand”, “go” and “rest”, respectively. The BMI environment was designed to passively open the patient’s paralyzed left hand during the movement imagination period as soon as MI-related ERD in the β-band was detected in the ispilesional, i.e., right, hemisphere (Walter et al., 2012; Gharabaghi et al., 2014a). The BMI algorithm was based on the spectral power values between 17 and 23 Hz for three selected channels (FC4, C4 and CP4). We selected these three electrodes to cover premotor, primary motor and somatosensory areas, all of which have been shown to be involved in functional restoration following stroke. However, the limited anatomical specificity of sensor-based EEG must be taken into consideration. The spectral power range was chosen on the basis of our previous findings (unpublished data) indicating that the effective corticospinal connectivity is mediated in a frequency-specific, amplitude- and phase-dependent way. We therefore applied the very same frequency-range and setup as in our previous BMI studies (Bauer et al., 2015; Gharabaghi et al., 2014a; Vukelić et al., 2014; Vukelić and Gharabaghi, 2015).

The spectral power was calculated using an autoregressive model order of 16 (McFarland and Wolpaw, 2008). This was fitted to the last 500 ms of the signal and updated every 40 ms. Orthosis-assisted movement was initiated or interrupted when five consecutive 40 ms epochs (i.e., 200 ms) were classified as ERD-positive or negative, respectively. An epoch was not regarded as ERD-positive until the output of the classifier exceeded a threshold θ (Walter et al., 2012; Gharabaghi et al., 2014a). The online signal processing was performed with the standard algorithm of the BCI2000 software (Mellinger and Schalk, 2007). With a bin width of 2 HZ and targeted bin centers of 18, 20 and 22 Hz the resulting frequency band was 17–23 HZ and corresponded to a wave length of between 43 and 59 ms. Choosing a data window of 500 ms enabled us to capture several cycles of these frequencies for reliable power analysis. This approach has already proved to be reliable in studies with the very same BMI setup (Walter et al., 2012; Bauer et al., 2015; Gharabaghi et al., 2014b; Vukelić et al., 2014; Vukelić and Gharabaghi, 2015).

We adjusted the threshold of the linear BMI classifier before each neurofeedback session. This adjustment was based on the recalculation of the neurofeedback parameters CRR, TPR and TNR/FPR of the previous session. Since the actual BMI performance of each session depended on the selected classifier threshold, the neurofeedback parameters had to be calculated independently of the used threshold. Hence, they had to be analyzed offline for different threshold levels (Bauer and Gharabaghi, 2015a,b).

### Data Acquisition and Analysis

Electroencephalogram (EEG) signals were recorded with BrainAmp DC amplifiers and an antialiasing filter (BrainProducts, Munich, Germany) from 32 Ag/AgCl scalp electrodes (sampling rate: 1000 Hz) in accordance with the international 10–20 system (FP1, FP2, F3, Fz, F4, FC5, FC3, FC1, FCz, FC2, FC4, FC6, C5, C3, C1, Cz, C2, C4, C6, CP5, CP3, CP1, CPz, CP2, CP4, CP6, P3, POz, P4, POz, O1, O2; reference: FCz, ground: AFz). Electrode impedances were maintained below 10 kΩ. Ambient noise may compromise the recordings as it often exceeds the frequency range of the physiological signals. To avoid an aliasing error due to undersampling of this noise, we made every effort to remove all potential sources of electrical noise from the experimental environment, i.e., high-frequency noise was actively avoided during the experiment and verified offline. Thanks to this approach, we do not observe high-frequency noise in our recordings (Bauer et al., 2015; Gharabaghi et al., 2014c; Vukelić et al., 2014; Vukelić and Gharabaghi, 2015). Furthermore, since our data analysis is based on the difference between two brain states (i.e., MI vs. rest), an unspecific noise would project into both conditions without affecting the difference.

During the experiment, surface electromyography (EMG) of the extensor digitorum communis (EDC) and flexor digitorum superficialis (FDS) muscles was recorded (band-pass filter: 0.1–1000 Hz, sampling rate: 1000 Hz). Since EMG contamination is known to compromise EEG-based BMI training (Gharabaghi et al., 2014b), experienced examiners were trained to recognize these artifacts and instructed the patient to minimize them. Similar to previous studies with healthy subjects (Vukelić et al., 2014) and severely affected stroke patients (Ramos-Murguialday et al., 2012) the patient was also instructed to avoid blinking, chewing, and head and body compensation movements. Together with visual inspection and feedback by the examiner, this approach proved to be a feasible method to prevent alternative BMI control. In addition, the EEG data was reanalyzed offline, removing all artifacted trials.

EEG was reanalyzed offline using MATLAB (MathWorks, Inc., Natick, MA, USA) and the FieldTrip open source toolbox.^1^ The data was band-pass filtered (3–120 Hz). Artefacts were rejected on the basis of trial variance, yielding an average of 147 ± 25 (mean ± SD) trials per session. Time-frequency analysis (multitaper) was performed and resulted in a time resolution of 0.1 s and a frequency resolution of 1 Hz. Power spectrum was normalized to the mean spectral distribution of the 4 s pre-movement rest period of the session. Mean movement-related spectral perturbation (ERSP) of α-, β- and γ-frequencies of the feedback electrodes were calculated for each session. After excluding significant baseline shifts between sessions, ERSPs were normalized to the first session to capture changes across sessions as an indicator of operant conditioning.

The BMI CA was calculated for each session. An exponential function was fitted on CA data by

(6) $$y(x)=a*e^{-bx}+c,$$

where *x* represents the session number, *y* the CA performance of the session, *a* and *b* the weighting factors and *c* the asymptote. The fitted line represents the learning curve and the asymptote (*c*) serves as a measure of final performance. The same fitting was performed for the ERSP.

### Clinical Evaluation

To capture the behavioral specificity of the BMI intervention beyond a general priming effect for physiotherapy, a precise clinical evaluation of the motor improvement is essential. Previous pilot studies reporting a benefit of BMI interventions in comparison to control groups reported a compound score (Ramos-Murguialday et al., 2013) or a general score of the whole upper extremity (Pichiorri et al., 2015). When comparing task-specific motor outcome parameters, BMI intervention might even be less effective than dose-matched control training (Ang et al., 2014).

We therefore evaluated hand (i.e., finger), wrist and arm motor function separately by the Upper Extremity Fugl-Meyer assessment (UE-FMA; Fugl-Meyer et al., 1975). This was conducted by an experienced physiotherapist before and after the 20–day training intervention (Ramos-Murguialday et al., 2013). To ensure that our results were comparable to earlier studies, *coordination*, *speed* and *reflexes* were not taken into account. This resulted in a modified UE-FMA, previously named cFMA (Ramos-Murguialday et al., 2013). Each score for the hand (i.e., finger), wrist and arm function, i.e., the parts (C), (B) and (A) of the UE-FMA, were provided separately and not as a compound score. Notably, an earlier controlled proof-of-concept study had fused the scores for hand (i.e., finger) and wrist function, i.e., the parts (C) and (B) of the UE-FMA, and termed the resulting compound value as the “hand” score (hFMA; Ramos-Murguialday et al., 2013). This might be misleading, given that the BMI intervention applied in both the earlier and present study trained the opening and closing of the *fingers* and *not wrist* movement. Reporting a compound “hand” score, i.e., summing up parts (C) and (B) of the UE-FMA, might therefore obscure a differentiation between the contribution of the BMI intervention and the rather unspecific effects of the subsequent physiotherapy applied in both the previous and the present report. To preserve specificity, we adhere to the original differentiation between hand (i.e., finger), wrist and arm function (Fugl-Meyer et al., 1975). However, for the sake of clarity, we name them finger (fFMA), wrist (wFMA) and arm (aFMA) function. The patient’s performance was video-taped and then, following the period of intervention, rated independently by five trained evaluators who were blinded to the intervention (pre/post evaluation).

## Empirical Dataset

A 68-year-old patient had suffered an ischemic cortico-subcortical stroke of the right hemisphere 34 months prior to his participation in the study. This had resulted in a persistent paresis of his left upper extremity with no volitional hand opening or finger extension (Medical Research Council motor scale <2). We tested the feasibility of reinforcement learning by controlling a robotic orthosis attached to the paralyzed hand of this chronic stroke survivor with ipsilesional sensorimotor β-band ERD during kinesthetic MI. The ability and evolution of BMI control, as well as physiological changes, were recorded in the course of twenty training sessions with dynamic threshold adaptation to probe for frequency-specific operant conditioning of β-band oscillations paralleled by task-specific motor improvements. The study protocol was approved by the local ethics committee.

**Figure 3:**
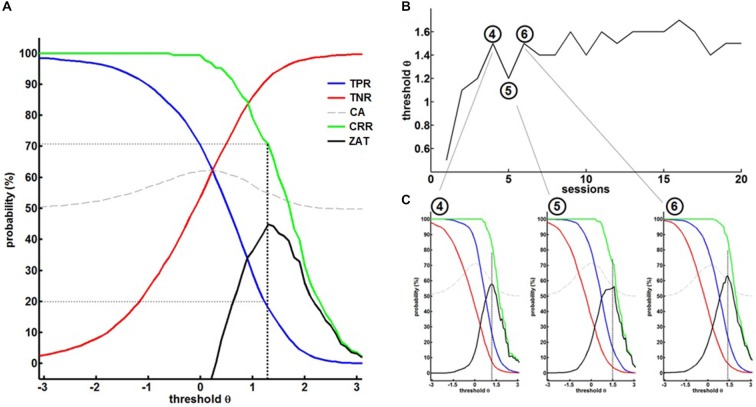
**(A)** Off-line re-analysis of the BMI data in the first session for different thresholds θ providing the true-positive rates (TPR), true-negative rates (TNR), classification accuracy (CA), correct response rate (CRR) and the zone of adjusted threshold (ZAT). ZAT peaks at *θ* = 1.2 corresponding to TPR = 20.9%, FPR = 9.9% and CRR = 72.2%. Hence, *θ* = 1.2 was selected for the succeeding BMI session. **(B)** Shows BMI thresholds in the course of training. **(C)** Exemplary off-line analysis after session 4, 5 and 6 illustrating on the evolution of BMI performance.

The first training session, i.e., the calibration session, started with a predefined, rather low threshold of *θ* = 0.5 to allow the patient to familiarize himself with the setup. The thresholds of the following sessions were adjusted according to the ZAT (see Figure 3A) which was robustly characterized by a TPR of about 20% and paralleled by a CRR of at least 70% and a TNR of above 90% (i.e., a FPR of below 10%).

This strategy allowed a dynamic adaptation of the threshold on the basis of the patient’s ability and course of learning (see Figure 3B). The fourth session, for example, was performed with a threshold *θ* = 1.5. The offline analysis (see Figure 3C left) suggested a threshold of *θ* = 1.2 for the next session. We therefore adjusted the threshold of the next, i.e., fifth session, to *θ* = 1.2. After training with this threshold, the offline analysis (see Figure 3C, center) revealed that the threshold could be increased to *θ* = 1.5 again. After adjusting the threshold of the next, i.e., sixth session, to *θ* = 1.5, offline analysis after training (see Figure 3C, right) showed that the threshold needed to be decreased, but this time to a threshold of *θ* = 1.4, revealing a learning effect in comparison to the fifth session (*θ* = 1.2). These stepwise adjustments led the patient to achieve higher thresholds, i.e., more β-band desynchronization, in the course of the training, thereby facilitating operant conditioning of the targeted brain state.

**Figure 4:**
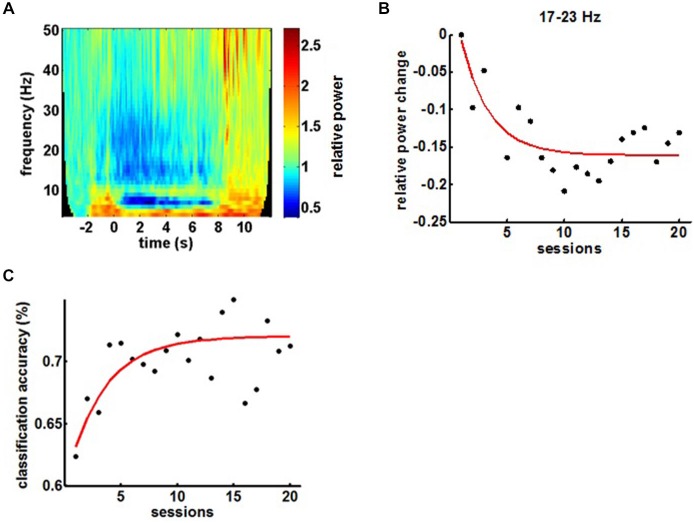
**(A)** Mean time-frequency plot of sensorimotor feedback electrodes (FC4, C4 and CP4) during a BMI training session showing the event-related spectral perturbations (ERSPs). **(B)** Evolution of β-ERD in the course of 20 feedback sessions. **(C)** Evolution of BMI control in the course of the training period.

BMI training induced the anticipated perturbations in the ipsilesional sensorimotor α (8–12 Hz)-, β (15–35 Hz)- and γ (>35 Hz)-frequency spectrum with ERD and ERS in the α/β- and γ-band, respectively (Figure 4A). Volitional β-ERD control of the EEG-based BMI enabled the chronic stroke survivor to open his paralyzed hand repetitively and reliably with the attached robotic orthosis. The β-ERD band, which was trained with feedback, changed significantly within the first 10 sessions and reached saturation in the course of the following 10 sessions, indicative of operant learning (Figure 4B). The BMI skill showed the very same exponential evolution in the first 10 sessions while reaching an asymptotic maximum in the following 10 sessions, thereby revealing a direct link to the modulated β-ERD (Figure 4C).

**Figure 5:**
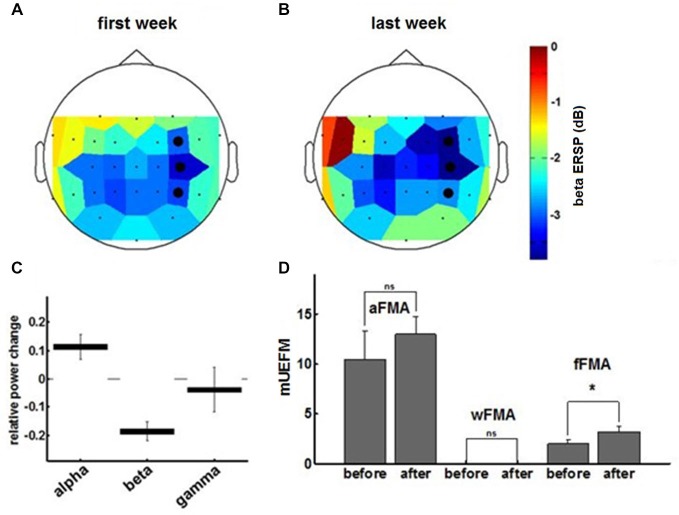
**The topoplot indicates the cortical distribution of β-ERD during the first (A) and final (B) training week.** The three feedback electrodes are shown as black dots. **(C)** Final ERSP changes for different frequency bands derived from the exponential fit to the ERSP learning curves at the end of training. Data represents mean ± 95%-confidence interval. **(D)** Fugl-Meyer assessment scores for arm, wrist and fingers prior to and subsequent to the training period.

Comparing α-, β- and γ-oscillations in the course of the intervention revealed frequency specificity for the β-band and, notably, a dissociation between the α- and the β-frequency band with regard to the evolution of the modulation range (Figures 5A,C). This β-ERD evolution was not restricted to the feedback electrodes (FC4, C4 and CP4) and included primary motor, premotor and supplementary motor areas of the ipsilesional cortex (Figure 5B). These physiological changes were paralleled by an overall clinical improvement of the upper extremity (cFMA: from 12.4 ± 3.1 to 16.2 ± 1.9, *p* = 0.049, Student’s *t*-test). However, disentangling the UE-FMA subscores revealed a task specificity of the functional gain with a significant improvement in the score related to the BMI trained finger movement (fFMA: from 2.0 ± 1.0 to 3.2 ± 0.45, *p* = 0.04) but not in the wrist (wFMA: from 0 ± 0 to 0 ± 0) and arm scores (aFMA: from 10.4 ± 2.88 to 13.0 ± 1.73, *p* = 0.121; Figure 5D).

## Discussion

Acquiring motor skills or re-learning them after brain injury, e.g., stroke, requires practice to induce motor learning (Doyon and Benali, 2005; Halsband and Lange, 2006). MI might also activate the sensorimotor system (Gao et al., 2011; Szameitat et al., 2012), thereby serving as an alternative training method (Halsband and Lange, 2006; Boe et al., 2014). The self-regulation of brain activity during MI can be supported by providing visual or proprioceptive feedback about the user’s current brain state using either BCI or BMI/BRI (Vukelić and Gharabaghi, 2015). First studies applying these approaches in stroke rehabilitation are promising (Prasad et al., 2010; Ang et al., 2011, 2014; Shindo et al., 2011; Ramos-Murguialday et al., 2013; Pichiorri et al., 2015). However, some conceptual and physiological questions remain as to the specificity of these interventions with respect to the achieved clinical gains. Controlled studies in both healthy subjects (Bai et al., 2014; Boe et al., 2014) and stroke patients (Ramos-Murguialday et al., 2013; Pichiorri et al., 2015) indicate that the adherence to MI training is higher with BCI/BMI technology than in interventions with sham or no feedback, i.e., that performing MI *effectively* is superior to performing it *ineffectively*. It is not surprising that such an *effective* MI intervention, when applied before physiotherapy, might increase the responsiveness of the brain to the subsequent physiotherapy like a priming mechanism, thereby improving the general clinical outcome (Ramos-Murguialday et al., 2013; Pichiorri et al., 2015). However, such BMI interventions have not achieved clinical benefits beyond those of dose-matched robot-assisted therapy (Ang et al., 2010, 2014) which has, in turn, provided only little additional benefit over dose-matched classical physiotherapy so far (Kwakkel et al., 2008; Lo et al., 2010; Klamroth-Marganska et al., 2014).

However, BMI technology sparks hopes that functional gains beyond this practice of *primed* and *intensive* physiotherapy might be achievable, particularly for the majority of stroke patients who still lack useful restoration of arm and hand function for activities of daily living despite rigorous rehabilitation training.

Operant conditioning of neural activity with BCI/BRI neurofeedback, e.g., challenging the patient to attain specific brain states that modulate corticospinal connectivity, is currently one favored neurophysiological concept to facilitate motor recovery (Daly and Wolpaw, 2008; Bauer and Gharabaghi, 2015b). Beta-band oscillations activity (15–30 Hz) over the sensorimotor cortex is particularly appropriate for this approach (Gharabaghi et al., 2014a,b,c; Vukelić and Gharabaghi, 2015) since it mediates the natural communication between cortex and peripheral muscular activity (Riddle and Baker, 2005; Witham et al., 2011; Davis et al., 2012; Kilavik et al., 2013), reflects sensorimotor control (Brittain et al., 2014), motor learning (Herrojo Ruiz et al., 2014; Pollok et al., 2014), corticospinal excitability (Takemi et al., 2013a,b), and the extent of functional impairment after stroke (Rossiter et al., 2014).

Recent studies in healthy subjects revealed that BCI feedback of MI-associated β-oscillations increased both laterality (Boe et al., 2014) and movement-associated desynchronization of the targeted β-frequency band (Bai et al., 2014) after three and five training sessions, respectively. Only one BRI session with proprioceptive feedback of MI-associated β-oscillations proved sufficient to activate a distributed cortical network (Vukelić et al., 2014) thereby bridging the gap between the abilities and cortical networks of MI and motor execution (Bauer et al., 2015). Moreover, a direct comparison between BCI and BMI/BRI feedback in the β-band and their neural oscillatory signatures revealed that closing the sensorimotor loop with proprioceptive feedback was superior to visual feedback only in supporting self-regulation of β-activity and activating a distinct cortical network resembling the natural activation during overt movement (Vukelić and Gharabaghi, 2015). Moreover, repetitive pairing of MI-related cortical activity and afferent input increased corticospinal excitability in healthy subjects (Mrachacz-Kersting et al., 2012; Niazi et al., 2012; Gharabaghi et al., 2014a; Xu et al., 2014).

Similar evidence for the neurophysiology of BCI/BMI training in stroke patients is sparse (Buch et al., 2012; Pichiorri et al., 2015). Surprisingly, even the study with the largest clinical gains of all pilot studies, in which BCI neurofeedback was provided prior to physiotherapy, showed no progress in the neurofeedback performance despite 4 weeks of training with 12 feedback sessions (Pichiorri et al., 2015). This might be related to the fact that an individualized selection of feedback channels and frequency bands was applied to maximize the differentiation between MI and rest and/or to the fixed classifier threshold throughout the course of training; an approach that is also chosen by most other BCI/BMI approaches. Moreover, the clinical benefit of the combination of BCI and MI as an add-on to standard physical therapy was not confined to the BCI tasks, i.e., grasping and finger extension, or even to the targeted upper limb function but also affected general clinical outcome scales (Pichiorri et al., 2015). This observation, while—from a clinical perspective—highly beneficial for those subacute stroke patients participating in this primed physiotherapy, nonetheless somewhat clouds the neurophysiological mechanisms related to the neurofeedback intervention. However, it is worth pointing out that, when comparing early and late training sessions in this study, only low β-band oscillations revealed a significant difference in their desynchronization pattern associated with BCI-MI. In the absence of any improvement in the BCI neurofeedback performance, this physiological pattern could most intuitively be interpreted as a correlate of the achieved behavioral gain following primed physiotherapy. This finding would corroborate the β-frequency band as a target substrate for the neurofeedback itself, particularly because its impaired activity range was also recently correlated to the extent of functional impairment after stroke (Rossiter et al., 2014).

It is tempting to relate the missing learning of the neurofeedback task, i.e., the lack of operant conditioning, to the pathological condition of the stroke patients. However, healthy subjects also often present with a large variability or even inability of brain self-regulation, referred to as BCI illiteracy (Vidaurre and Blankertz, 2010). A more general misalignment between the subject’s abilities and the classifier/neurofeedback strategy therefore has to be assumed. This has led to attempts to estimate the participant’s cognitive resources and the instructional efficacy of neurofeedback on the basis of BCI/BMI performance measures, revealing that particularly participants with low abilities for brain self-regulation, e.g., stroke patients with a reduced beta-band modulation range, might present a broad *zone of learning* when aligning the task difficulty with their ability (Bauer and Gharabaghi, 2015a).

Compared to studies with healthy subjects in a limited number of neurofeedback sessions, stroke studies usually span several weeks with a comparably high number of training sessions. This poses a special challenge to keep up the motivation of the participants and their adherence to the task even when feedback is provided. Optimizing metabolic cost of BCI/BMI control during long-term application (Jackson and Fetz, 2011) might be one possible explanation for the lack of operant conditioning. Therefore, BCI/BMI tasks need to provide incentives to achieve higher levels of the targeted brain state (Carmena, 2013), and, in turn to achieve frequency- and task-specific effects of the intervention (Zoefel et al., 2011).

Bayesian modeling of neurofeedback, particularly in long-term interventions with many iterations, indicates that operant conditioning might be optimized when an adaptation strategy for threshold selection, i.e., the difficulty level of the feedback devise, is applied in the course of the training balancing challenge and reward (Bauer and Gharabaghi, 2015b). The neurofeedback strategy proposed here for motor restoration after stroke provides evidence for the first time that dynamic threshold adaptation based on reinforcement learning may lead to frequency-specific operant conditioning of β-band oscillations paralleled by task-specific motor improvements. The reported observations—albeit in one patient and therefore requiring investigation in a larger cohort—might, however, inform the design of further studies in this field.

Since the presented concept achieved a continuous and significant BMI improvement in the very first sessions and provided a direct brain-behavior relationship with functional gains specific for the BMI trained task, it might inspire future research in this direction. Such studies would need to directly compare different feedback targets, e.g., β-band vs. α-band oscillations, and to relate respective changes of the oscillatory range to distinct functional gains using specific scores and measures. Moreover, the classical BCI/BMI concept of maximizing CA of the decoding algorithms needs to be complemented by and/or compared to approaches that adjust the feedback in the course of the training period to account for the cognitive load and the learning experience of the participant.

When patients do not gain volitional control of β-band oscillations via a standard EEG-based approach despite the strategies mentioned above—e.g., due to an extended cortical lesion and distorted physiology—epidural recordings of field potentials may nonetheless facilitate the detection and neurofeedback training of this physiological target (Gharabaghi et al., 2014b). Such an approach closer to the neural signal source may also induce clinical gains after a shorter therapy time than is usually applied with the standard EEG technique (Gharabaghi et al., 2014c). Furthermore, the frequency-specific increase of β-ERD not only in the primary motor cortex, but also in medial premotor and supplementary motor areas of the ipsilesional cortex, suggests that these latter regions are a potential target for future neurorestorative interventions in stroke patients on the basis of neurofeedback and/or brain stimulation (Plow and Machado, 2014).

The present case study showed a specific and significant improvement in the finger function (in the finger part [C] of the UE-FMA score) trained with the BMI intervention (i.e., task-specificity). However—as in other BMI studies with severely affected stroke patients (Ramos-Murguialday et al., 2013)—these gains did not lead to relevant improvements of activities of daily living that necessitate hand opening. To achieve substantial gains for the paralyzed hand, more extended training periods than those currently applied, i.e., beyond 4 weeks, might be necessary, since time in therapy is a robust predictor of recovery across different intervention types, at least for less severely affected stroke patients with residual hand function (Lohse et al., 2014).

However, such modifications of time scheduled for training or actual practice time might not suffice *per se* to pass the critical threshold for clinically meaningful improvements of hand function. Additional interventions, such as concurrent state-dependent brain stimulation (Gharabaghi et al., 2014a) to restore or unmask latent corticospinal connectivity in a functionally effective way, may be required.

Preliminary evidence suggests that even severely affected stroke patients lacking residual hand function have the potential to train and extend their β-modulation range and achieve specific motor gains when receiving neurofeedback training based on concepts of reinforcement learning. If applied for a sufficient time and at the appropriate intensity, this intervention may in itself constitute a therapeutic approach for functional restoration.

## Conflict of Interest Statement

The authors declare that the research was conducted in the absence of any commercial or financial relationships that could be construed as a potential conflict of interest.

## Footnotes

^1^http://fieldtrip.fcdonders.nl/
